# Enhanced herbicidal activity of coumarin via carbon dot nanoformulation: synthesis and evaluation

**DOI:** 10.1002/ps.8782

**Published:** 2025-03-31

**Authors:** Yuelan Yin, Jingnan Zou, Zhichao Chen, Yihu Yang, Caixia Wu

**Affiliations:** ^1^ College of Animal Science and Technology Yangzhou University Yangzhou China

**Keywords:** coumarin, carbon dots, nanomaterials, bioactivity

## Abstract

**BACKGROUND:**

The integration of nanocarriers with biochemicals can markedly enhance the stability and efficacy of these agents, which will help to diminish the reliance on chemical pesticides, and contribute to the advancement of sustainable agricultural practices.

**RESULTS:**

We prepared nanometer carbon dots (CDs) from black wolfberry (*Lycium ruthenicum* Murr.) and synthesized coumarin‐loaded carbon dots (Cm‐CDs) nanocomposites. The CDs exhibited a uniform distribution and high stability, with a coumarin loading rate of 65.45%. Experimental results on the herbicidal activity of both coumarin and Cm‐CDs against *Portulaca oleracea* and *Setaria viridis* showed that, compared with coumarin alone, Cm‐CDs completely inhibit the germination of *S. viridis* or prevent further development of *P. oleracea* post‐germination at lower concentrations. The application of Cm‐CDs has the potential to significantly diminish the biomass of *P. oleracea* and *S. viridis* (the dry weight decreased by 69.3% and 63.61% respectively), leading to a cessation of growth and the manifestation of wilting symptoms (*P* < 0.05). This indicates that the binding of coumarin to CDs markedly enhances the herbicidal efficacy of coumarin. Both coumarin and Cm‐CDs exhibit consistent alterations in biomass, hormone levels, antioxidant enzyme activity, malondialdehyde (MDA) content, root morphology, and vitality, however, the effects observed with Cm‐CDs were consistently more pronounced than those associated with coumarin (*P* < 0.05).

**CONCLUSION:**

In both the germination bioassay and the pot experiment, Cm‐CDs demonstrated stronger herbicidal toxicity. Both coumarin and Cm‐CDs exhibit identical modes of action on *P. oleracea* and *S. viridis*. This study confirms that CDs can serve as effective nanocarriers to markedly enhance the herbicidal biological activity of coumarin in controlled conditions. © 2025 The Author(s). *Pest Management Science* published by John Wiley & Sons Ltd on behalf of Society of Chemical Industry.

## Graphical abstract

We synthesized carbon nanocarriers from black wolfberry and combined them with coumarin to prepare coumarin‐loaded carbon dots (Cm‐CDs) nanocomplexes, which could significantly enhance the inhibitory effect of coumarin on weeds.

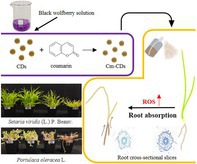

## 1 INTRODUCTION

In recent decades, the application of chemically synthesized pesticides for controlling weeds, pests, and plant diseases in agricultural fields has achieved significant success.^1^, ^2^, ^3^ However, this practice has also led to serious ecological and environmental challenges as well as concerns over food safety. Botanical pesticides offer several advantages, including ease of material acquisition, favorable environmental compatibility, strong selectivity, and low resistance evolution. Given the increasing demand for environmental protection, these natural alternatives are increasingly recognized as viable substitutes for chemical pesticides. Among botanical pesticides, coumarin stands out as a crucial allelopathic compound commonly found in higher plants, such as those from the *Rutaceae* and *Apiaceae* families, as well as in certain microorganisms.

Studies have demonstrated its efficacy in controlling pests, pathogens, and weeds in agricultural environments.^4^, ^5^, ^6^ For example, coumarin natural compounds can induce neurotoxic effects on pests,^7^ attack pathogens through multi‐target mechanisms,^8^ induce systemic acquired resistance in plants,^9^ and exhibit allelopathic and ecological regulatory functions.^10^ Natural extracts usually contain multiple active ingredients that can act synergistically on different targets^11^ and exert their effects by inducing host immunity rather than direct killing, thereby reducing the risk of resistance development under strong selection pressure. Some coumarins have already been utilized in agricultural production and recognized as promising alternatives to conventional chemical pesticides. For example, coumarin substance osthole, a biological pesticide with insecticidal and antibacterial activity, was first patented as a pesticide in 2003 and was provisionally registered as a pesticide in the same year.^12^ In 2017, a 1% osthole emulsion product was approved for registration, primarily targeting wheat powdery mildew.^13^

However, the limited availability, low concentration levels, and high extraction costs of plant‐derived coumarin currently hinder its widespread application in practical production. Consequently, enhancing the biological activity and utilization efficiency of coumarin remains a critical challenge that must be addressed. The development of innovative nanocarriers combined with plant‐derived pesticides represents an effective strategy to improve the efficacy of these natural products.

As an emerging nanomaterial, carbon dots (CDs) have gained significant traction in bioimaging, medicine, and chemical analysis due to their advantageous properties, including non‐toxicity, biocompatibility, and ease of loading.^14^ Biomass‐derived CDs exhibit low toxicity, eco‐friendliness, and controllability, making them highly suitable as carriers for pesticides. In the future, they are expected to become a primary delivery system for new green pesticides. Research has demonstrated that CDs can serve as effective carriers to enhance the stability and solubility of active pharmaceutical ingredients while improving the targeted delivery of drug molecules.^15^ As drug carriers, CDs not only increase the stability of pharmaceuticals but also enhance the solubility of hydrophobic compounds in aqueous environments. Furthermore, they facilitate the aggregation and targeted delivery of drugs.^16^ Nanoformulations based on CDs can minimize drug loss, thereby improving the effective utilization rate and enhancing therapeutic efficacy.^17^

In this study, biomass‐derived CDs were developed and combined with coumarin to create a coumarin nanocomposite. This approach aims to enhance the biological activity and utilization efficiency of coumarin. The synthesized coumarin‐loaded carbon dots (Cm‐CDs) nanocomposite was applied to two weed species, *Portulaca oleracea* and *Setaria viridis*, to evaluate its herbicidal activity. The differences in efficacy and mode of action between the nanocomposite and direct application of coumarin were compared to validate the biological activity and potential applications of the coumarin nanocomposite. This study provides valuable insights into the preparation and use of CDs‐based biopesticides and contributes to the development of novel products in this field.

## 2 MATERIALS AND METHODS

### 2.1 Preparation of carbon dots (CDs)

**Figure 1:**
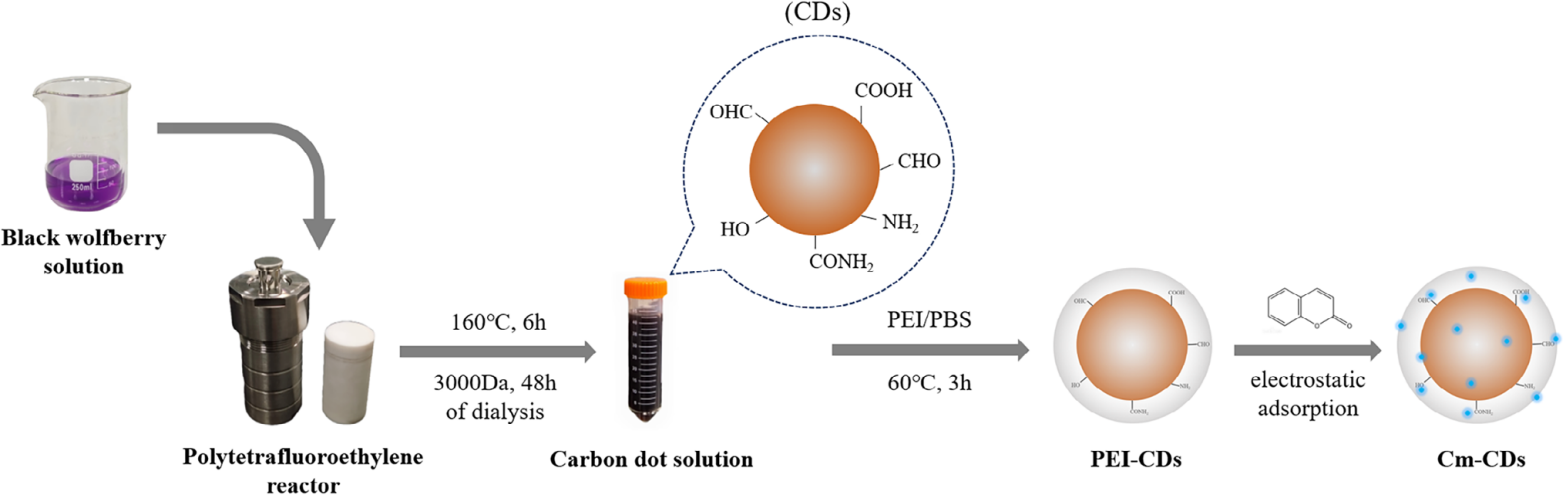
Flow chart of the preparation of coumarin‐loaded carbon dots (Cm‐CDs) nano drug delivery system.

In this study, fluorescent CDs were synthesized using a bottom‐up hydrothermal method.^18^ The preparation process is illustrated in Fig. 1.

Initially, 2 g of black wolfberry were weighed and placed into a 50 mL beaker. Deionized water was added to reach the 50 mL mark, and the mixture was soaked for 12 h. The solution was then filtered to remove residual black wolfberry material. The resulting filtrate was transferred to a polytetrafluoroethylene hydrothermal reactor, which was placed in an oven at 160 °C for 6 h. After the reaction, the reactor was allowed to cool naturally to room temperature. A filter head was used to remove impurities with larger diameters, and the solution was dialyzed in a 3000 Da dialysis bag for 24 h to obtain a purified fluorescent CDs solution. Finally, the fluorescent quantum CDs were acquired through rotary evaporation and vacuum drying.

### 2.2 Characterization of carbon dots (CDs)

#### 2.2.1 Morphological analysis

The morphology of the CDs was characterized using transmission electron microscopy (TEM).

#### 2.2.2 Infrared spectrum analysis

The prepared solid CDs sample was mixed with spectroscopically pure potassium bromide (KBr) at a ratio of 1:100 to 1:200. The mixture was thoroughly ground to ensure homogeneity and pressed into a transparent pellet. The pellet was then placed in the sample chamber of a Cary 610/670 micro‐infrared spectrometer for spectral scanning to obtain the infrared spectrum.

#### 2.2.3 Fluorescence spectral properties

The fluorescence spectra of the CDs were measured using a fluorescence spectrophotometer. Scanning was performed at various excitation and emission wavelengths under detection conditions set at sensitivity level 5 with low‐speed scanning to determine the optimal excitation and emission wavelengths. Under identical conditions, varying amounts of coumarin were added, and the corresponding changes in fluorescence intensity were recorded.

### 2.3 Preparation and optimization of coumarin‐loaded carbon dots (Cm‐CDs) nanodrug delivery system

#### 2.3.1 Preparation of PEI/PBS buffer reagent mixture

To prepare the PEI/PBS buffer reagent mixture, 0.5 g of polyethyleneimine (PEI) was dissolved in 100 mL of phosphate‐buffered saline (PBS) buffer, ensuring the pH was between 7.2 and 7.4. Next, 0.25 g of CDs powder was added to this solution and fully dissolved using ultrasonic treatment. Subsequently, the mixed solution was placed in a constant temperature water bath at 60 °C and stirred for 3 h to obtain the PEI‐CDs mixed solution.

#### 2.3.2 Preparation of coumarin‐loaded carbon dots (Cm‐CDs) complex

**Figure 2:**
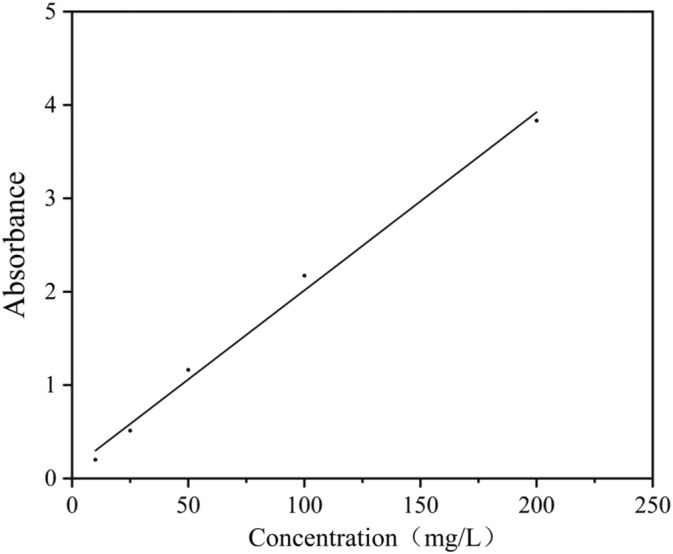
Standard curve of the relationship between coumarin concentration and absorbance.

A coumarin solution with a concentration of 0.1 g/L was combined with the prepared PEI‐CDs mixed solution and shaken thoroughly at room temperature. The ultraviolet (UV)‐visible absorption characteristic peak for coumarin occurs at 345 nm, where its absorption value exhibits a strong linear correlation with concentration. The absorption values of coumarin solutions at various concentrations were measured at 345 nm to construct a standard curve (Fig. 2). A volume of 3 mL of coumarin solution was mixed with CDs solutions in varying volumes to achieve carrier‐to‐drug mass ratios of 0.5, 1, 1.5, 2, 2.5, 3, and finally up to 4 respectively, these mixtures were stirred overnight at room temperature to ensure complete interaction between the coumarin and the PEI‐CDs.

#### 2.3.3 Dialysis and drug loading rate calculation

Subsequently, 5 mL of each mixed solution was transferred into a dialysis bag and immersed in a beaker containing 50 mL of deionized water for 24 h. After dialysis, 200 μL of the external solution was sampled to measure its absorbance. The unbound coumarin content was calculated using the standard curve. The drug loading rate (%) was determined using the following formula:

$$\text{Drug loading rate}\left(\%\right)=\begin{array}{l} \left(\text{Quantity of bound drug}/\text{Quantity of nanocarrier}\right)\times 100. \end{array}$$

### 2.4 Effects of coumarin and coumarin‐loaded carbon dots (Cm‐CDs) on weed growth

Seeds of *P. oleracea* and *S. viridis* were surface‐sterilized by immersion in a 10% sodium hypochlorite solution. The seeds were soaked and agitated for 5 to 10 min, followed by thorough rinsing with distilled water for subsequent use. Next, 30 *P. oleracea* seeds and 20 *S. viridis* seeds were placed in a 9 cm diameter culture dish lined with qualitative filter paper. Solutions of coumarin, CDs, and Cm‐CDs at varying concentrations (5 mL each) were added to ensure complete saturation of the weed seeds. Each treatment was replicated three times, while deionized water without any agents served as a blank control. The results indicated that both coumarin and Cm‐CDs exhibited significant inhibitory effects on the germination of weed seeds.

Subsequently, a pot experiment was conducted. Seeds of *P. oleracea* and *S. viridis* were evenly sown in No. 7 pot and cultured in distilled water for 5 days at a temperature of 28 °C, with 60% humidity and a 12 h:12 h light/dark photoperiod (LED growth lamp intensity: 8000 lx). Solutions of coumarin, CDs, and Cm‐CDs were prepared with concentration gradients of 0.2, 0.5, 0.8, 1.2, and 1.5 g/L, with deionized water serving as the control. The plants were sprayed daily for three consecutive days, with each treatment replicated three times. A preliminary biomass assessment was conducted on the weeds following the various treatments.

### 2.5 Determination of toxicity of coumarin to weeds

Seeds of *P. oleracea* and *S. viridis* were surface‐sterilized by immersion in a 10% sodium hypochlorite solution. The seeds were soaked and stirred for 5–10 min, followed by thorough rinsing with distilled water. Subsequently, the seeds were transferred to a culture dish lined with cotton and gauze, moistened with deionized water, and soaked for 12 h in darkness until the seeds turned white.

Gradient concentrations of coumarin solutions (10, 25, 50, 75, 100, and 120 mg/L) were prepared using deionized water. Then, 25 weed seeds of uniform size and consistent whitening were selected and placed in a 12 cm diameter culture dish lined with two qualitative filter papers. Each dish was treated with 9 mL of the respective coumarin solution to fully saturate the seeds. Each concentration was replicated five times, with deionized water serving as a blank control.

The treated culture dishes were incubated in a light incubator at 28 °C for 3 days, followed by an additional 4 days under photoperiod conditions of 14 h:10 h light/dark (LED growth lamp intensity: 8000 lx). After 7 days of treatment with various concentrations, the root lengths of *P. oleracea* and *S. viridis* were measured, and the inhibition rates were calculated. A toxicity regression equation was established to determine the median inhibitory concentration (IC_50_) of coumarin on root lengths for both species.

### 2.6 Determination of plant physiological indicators

A coumarin solution was diluted to achieve a concentration corresponding to its IC_50_ value. The Cm‐CDs solution was then prepared according to the calculated optimal mass ratio to ensure that the effective concentration of coumarin in both the coumarin solution and the Cm‐CDs solution remained consistent. Deionized water (as a control) and CDs solution were utilized as controls during testing, which was conducted using a plate method. The soluble protein content was quantified using the Coomassie Brilliant Blue G‐250 method,^19^ soluble sugar content was assessed via the anthrone colorimetric method,^20^ subsequently, α‐amylase activity was determined from the solid residue obtained after removing soluble sugars.^21^

For enzyme extraction, 0.2 g of treated weed seeds were weighed and placed into a pre‐cooled mortar. A total of 2 mL of pre‐cooled PBS buffer (pH 6.0, 0.1 mol/L) was added, and the mixture was homogenized in an ice bath. The homogenate was transferred to a 2 mL centrifuge tube and centrifuged at 4 °C for 20 min at 12 000 rpm. The resulting supernatant was collected as the crude enzyme solution and stored in a refrigerator for subsequent analysis.

The activity of superoxide dismutase (SOD) was assessed using the nitroblue tetrazolium (NBT) photoreduction method,^22^ peroxidase (POD) activity was measured by the guaiacol method,^23^ catalase (CAT) activity was determined through visible light spectrophotometry.^24^ Additionally, malondialdehyde (MDA) content was quantified using the thiobarbituric acid (TBA) method.^25^

The abscisic acid (ABA) and gibberellin (GA3) enzyme‐linked immunosorbent assay (ELISA) kits, provided by Shanghai Qiaodu Biotechnology Co., Ltd (Shanghai, China), were used to quantify plant hormone content in accordance with standard procedures for ELISA in basic biological science experiments.

### 2.7 Comparison of the effects of coumarin and its nanoformulation on inhibiting root development of weeds

In this experiment, a hydroponic method was employed. Weed seeds exhibiting consistent growth were selected for germination under dark conditions at 28 °C. Once the seeds developed white shoots, the seeds were transplanted into a sponge box saturated with deionized water and subsequently placed into a hydroponics chamber. The culture was maintained in a controlled environment with day and night temperatures set at 26 °C and 22 °C, respectively, a 12 h:12 h light/dark cycle, and relative humidity maintained between 60% and 70%. The control group was treated with water only. Treatment groups included coumarin solution at moderate inhibitory concentrations for *P. oleracea* and *S. viridis*, as well as Cm‐CDs solution at equivalent effective concentrations. After 7 days of treatment, root samples were collected for analysis. The relative electrical conductivity of the root system was measured by the soaking method, the root activity was measured by the TTC staining method (2,3,5‐triphenyltetrazolium chloride staining method) and paraffin sections of roots were prepared by Sewell Testing Ltd (Jiaxing City, Zhejiang Province, China).^26^, ^27^

### 2.8 Data analysis

Statistical analysis of the data was performed using Microsoft Excel 2016 and PASW Statistics 18, while graphical representations were generated using Origin 2018 and GraphPad Prism 9.5.0.

## 3 RESULTS

### 3.1 Characterization results of carbon dots (CDs)

**Figure 3:**
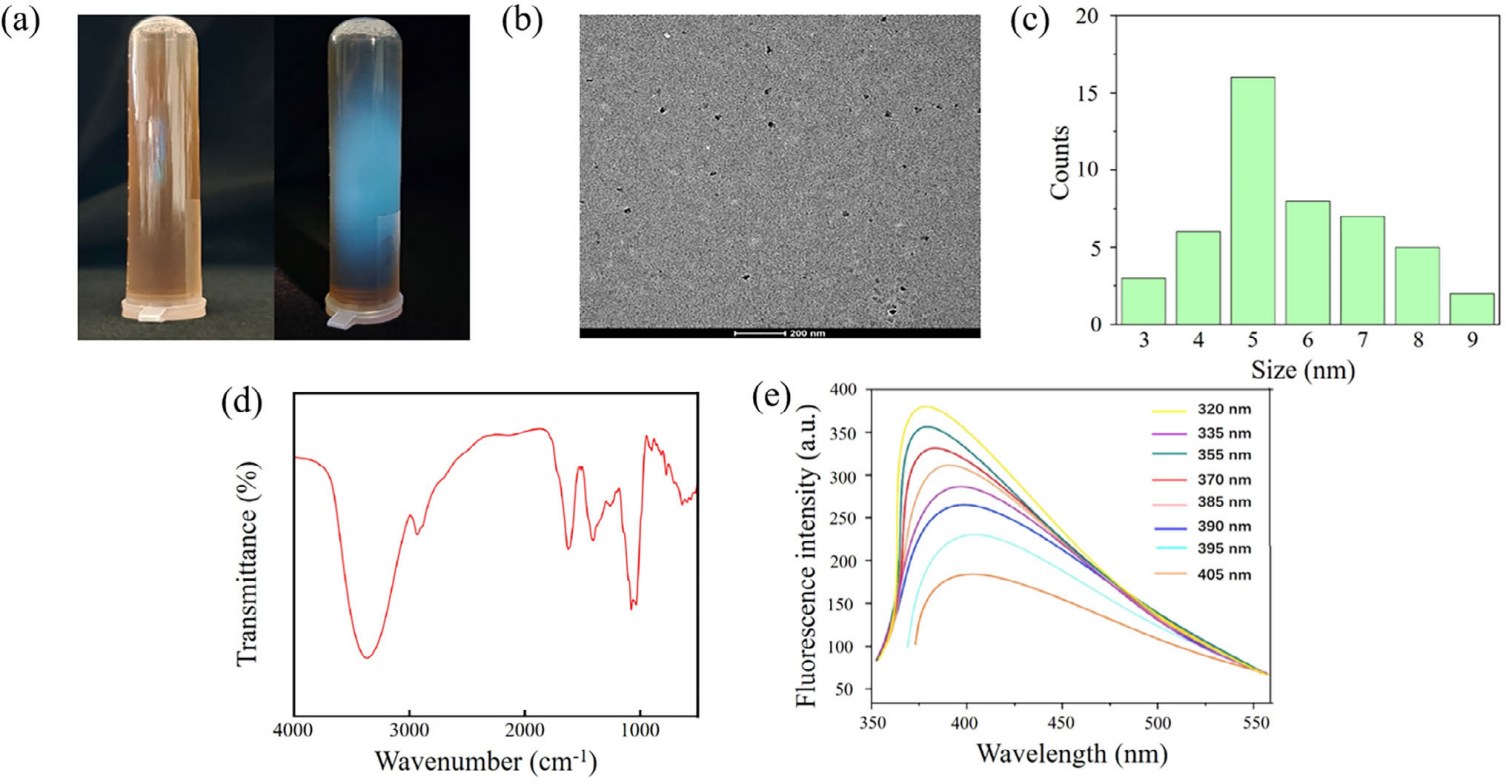
(a) Comparison of the appearance of carbon dots (CDs) solution under sunlight and 365 nm UV light. (b) Transmission electron microscopy image of CDs. (c) Statistical graph of CDs particle size distribution. (d) Infrared spectrum of CDs. (e) Fluorescence spectra of CDs at different wavelengths.

Under optimal preparation conditions, a stable brown CDs solution was successfully obtained. Upon irradiation with a 365 nm UV lamp, the CDs solution was subjected to comparative analysis, as shown in Fig. 3(a). The results indicate that the synthesized CDs exhibit excellent fluorescence characteristics. The morphology and particle size were characterized using TEM. A total of 50 CDs depicted in Fig. 3(b) were analyzed for particle size statistics. Due to the complex and diverse components of biomass itself, the size of the synthesized CDs is also different.^28^ The particle size distribution of the CDs is presented in Fig. 3(c), revealing that the sizes predominantly range from 3 to 9 nm. The particles are small, approximately spherical in shape, and demonstrate good dispersibility, with an average particle size of 5.71 nm.

The surface functional group structure of the CDs was analyzed using an infrared spectrometer, with the results presented in Fig. 3(d). Characteristic absorption peaks corresponding to the stretching vibration of –C=O appear at 1683 and 1350 cm^−1^, indicating the presence of carboxyl functional groups on the surface of the CDs. Additionally, characteristic absorption peaks for both stretching and bending vibrations of –OH were observed at 3358 and 1074 cm^−1^, respectively, suggesting a significant quantity of hydroxyl groups alongside carboxyl groups.

Furthermore, a peak associated with the –C–N stretching vibration was detected at 1472 cm^−1^, signifying the formation of a –CONR bond and suggesting the presence of amino (–NH_2_) groups on the CD surface. This analysis confirms that various functional groups including carboxyl, hydroxyl, and amino groups are located on the CD surfaces. These functional groups not only enhance hydrophilicity, thereby improving water solubility, but also provide numerous sites for potential surface modifications.

To investigate the optical properties of the CDs, we measured the changes in fluorescence intensity across a range of excitation wavelengths. As shown in Fig. 3(e), the peak emission wavelength of the CDs varies with increasing excitation wavelength, demonstrating an excitation‐dependent emission characteristic with known properties of CDs. This behavior is attributed to variations in particle size or passivation effects caused by different chemical species on the CD surface. Notably, as the excitation wavelength increases, the fluorescence intensity initially rises before declining, with the maximum emission wavelength determined to be 320 nm.

### 3.2 Establishment and optimization of coumarin‐loaded carbon dots (Cm‐CDs) nanodrug delivery system

**Figure 4:**
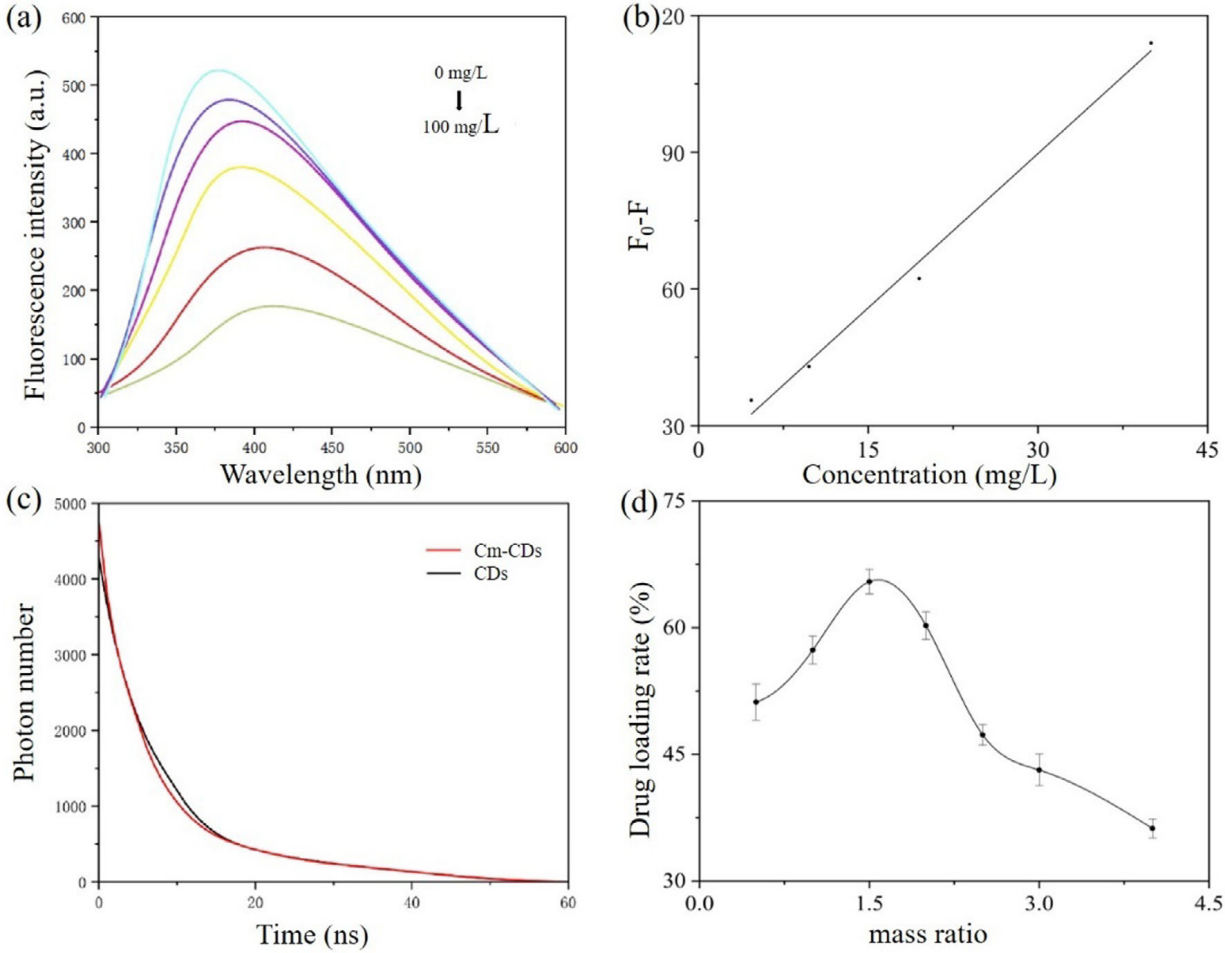
(a) Fluorescence response spectra of carbon dots (CDs) to coumarin at different concentrations. (b) Linear change of fluorescence intensity after coumarin at different concentrations binds to CDs. (c) Fluorescence lifetime curves of CDs and coumarin‐loaded carbon dots (Cm‐CDs). (d) Drug loading rate of Cm‐CDs at different mass ratios.

The results of Fig. 4(a) indicate that the fluorescence intensity of CDs diminishes as the concentration of coumarin increases. Furthermore, Fig. 4(b) demonstrates a strong linear relationship between the quenching intensity of CDs fluorescence and coumarin concentration within the range of 0–40 mg/L, exhibiting a correlation coefficient of 0.9928 and described by the linear equation *F*_0_ − *F* = 2.2569*x* + 22.012.

Fluorescence quenching refers to any process that reduces the fluorescence intensity of a fluorescent substance or disrupts its linear relationship with concentration. This phenomenon encompasses two mechanisms: dynamic quenching and static quenching. Dynamic quenching occurs through rapid collisions between fluorescent molecules and quencher, resulting in energy transfer from excited fluorescent molecules to quenchers and a consequent reduction in fluorescence intensity. In contrast, static quenching arises when fluorescent species and quenchers form non‐luminescent complexes through close‐range interactions.

The analysis of the fluorescence lifetime curves for CDs and Cm‐CDs, as shown in Fig. 4(c), indicates no shift in their profiles. This observation suggests that the fluorescence quenching is predominantly due to static quenching, providing compelling evidence for the interaction between CDs and coumarin.

Upon calculation, it was found that the drug loading rate of the Cm‐CDs system exhibited an initial increase followed by a decrease with rising mass ratios (Fig. 4(d)). The maximum drug loading rate of 65.45% was achieved at a mass ratio of 1:1.5. This phenomenon is attributed not only to the loading function of the CDs itself, but also to the strong electrostatic attraction between the substantial negative charges on the surface of CDs, resulting from deprotonation and the positive charges associated with coumarin. A higher drug loading rate correlates with a greater degree of interaction between CDs and coumarin, thereby enhancing their combined efficacy.

### 3.3 Effects of coumarin and coumarin‐loaded carbon dots (Cm‐CDs) treatments on weed growth

**Figure 5:**
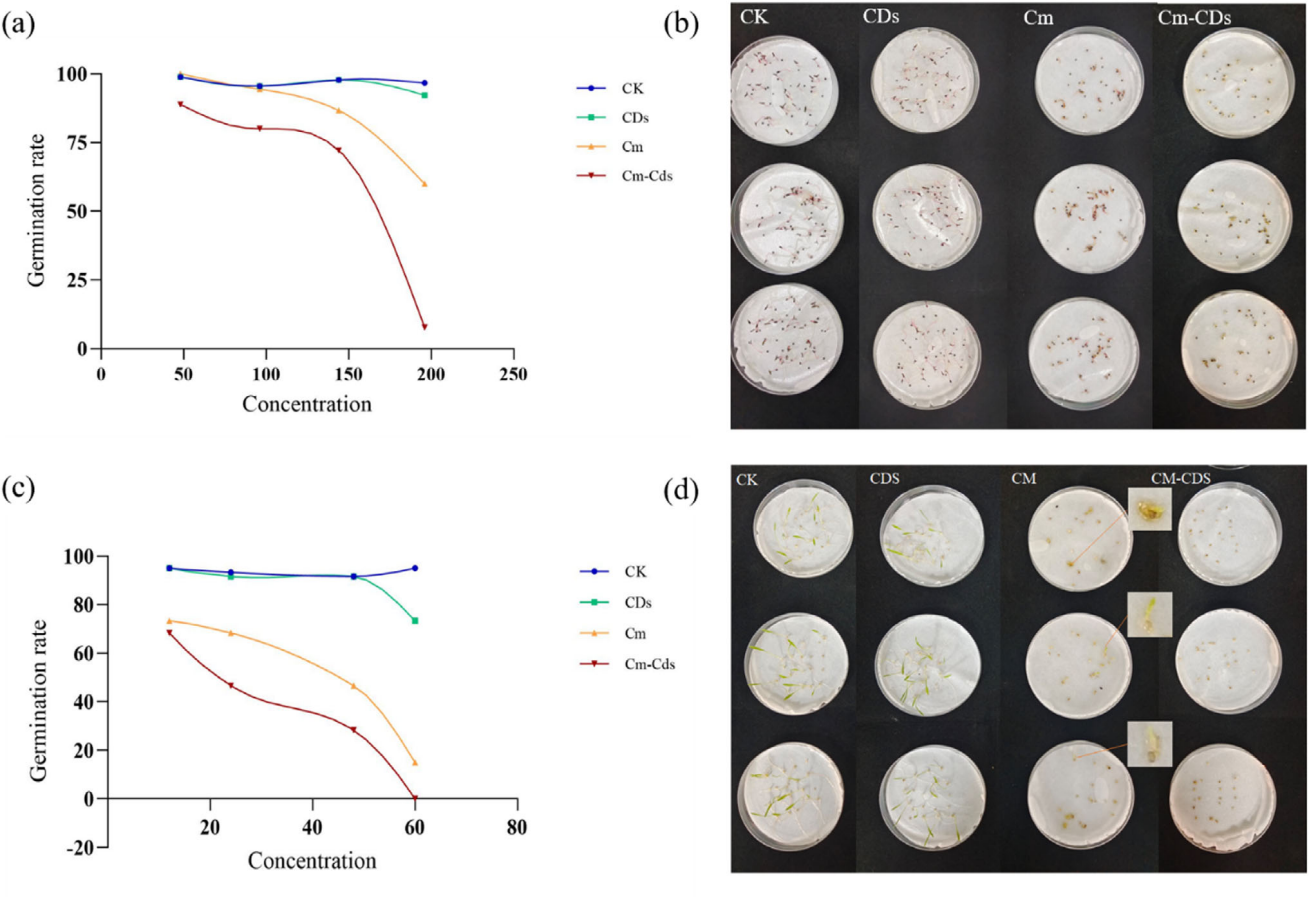
Effects of different treatments on the germination rates of *Portulaca oleracea* and *Setaria viridis*. (a) *Portulaca oleracea* germination rate. (b) Photograph of germination of *P. oleracea* 3 days after treatment with a 196 mg/L concentration reagent. (c) *Setaria viridis* germination rate. (d) Photograph of germination of *S. viridis* 5 days after treatment with a 60 mg/L concentration reagent. CK, control; CDs, carbon dots; Cm, coumarin; Cm‐CDs, coumarin‐loaded carbon dots.

As shown in Fig. 5, CDs exhibited negligible effects on the germination of both *P. oleracea* and *S. viridis*. In contrast, coumarin and Cm‐CDs significantly inhibited their germination (*P* < 0.05), with Cm‐CDs showing a stronger inhibition, specifically, it completely inhibited seed germination of *S. viridis* at a concentration of 60 mg/L. Due to the rapid germination characteristics of *P. oleracea* seeds, which readily break through their seed coat upon water absorption, complete inhibition was not achieved. However, buds treated with Cm‐CDs displayed darker coloration, shorter stature, thicker morphology, and slower growth rates.

**Figure 6:**
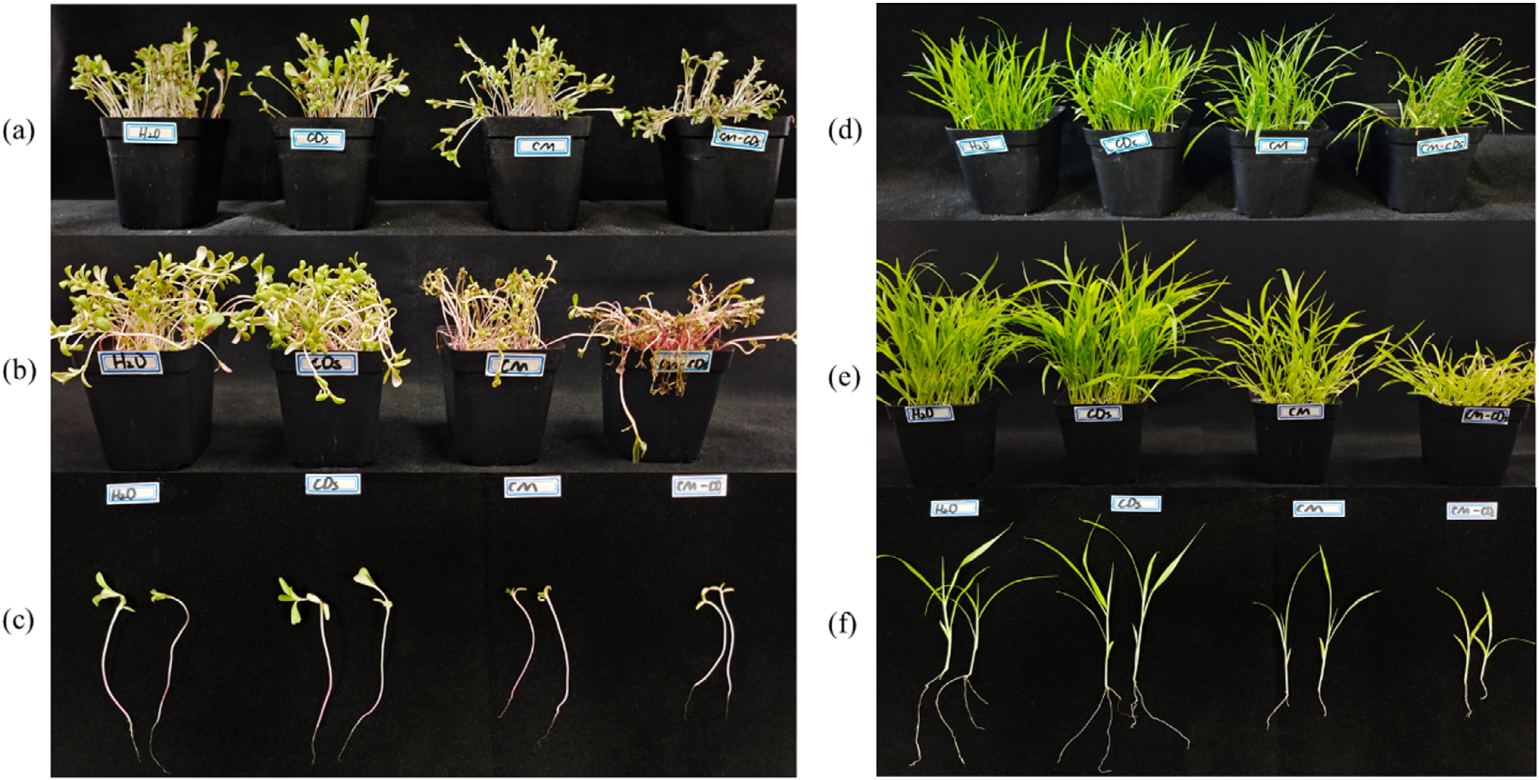
Effects of different agents on *Portulaca oleracea* (left) and *Setaria viridis* (right) after 3 days of spraying. (a), (d) Treatment concentration is 0.5 g/L; (b), (e) treatment concentration is 1.5 g/L; (c), (f) pictures of plant roots after treatment with 1.5 g/L concentration.

Figure 6 presents the results of pot experiments, showing that *P. oleracea* treated with coumarin and Cm‐CDs exhibited symptoms such as wilting and scorching. Similarly, *S. viridis* appeared stunted and yellowed, with the effects induced by Cm‐CDs being more pronounced than those caused by coumarin alone.

**Figure 7:**
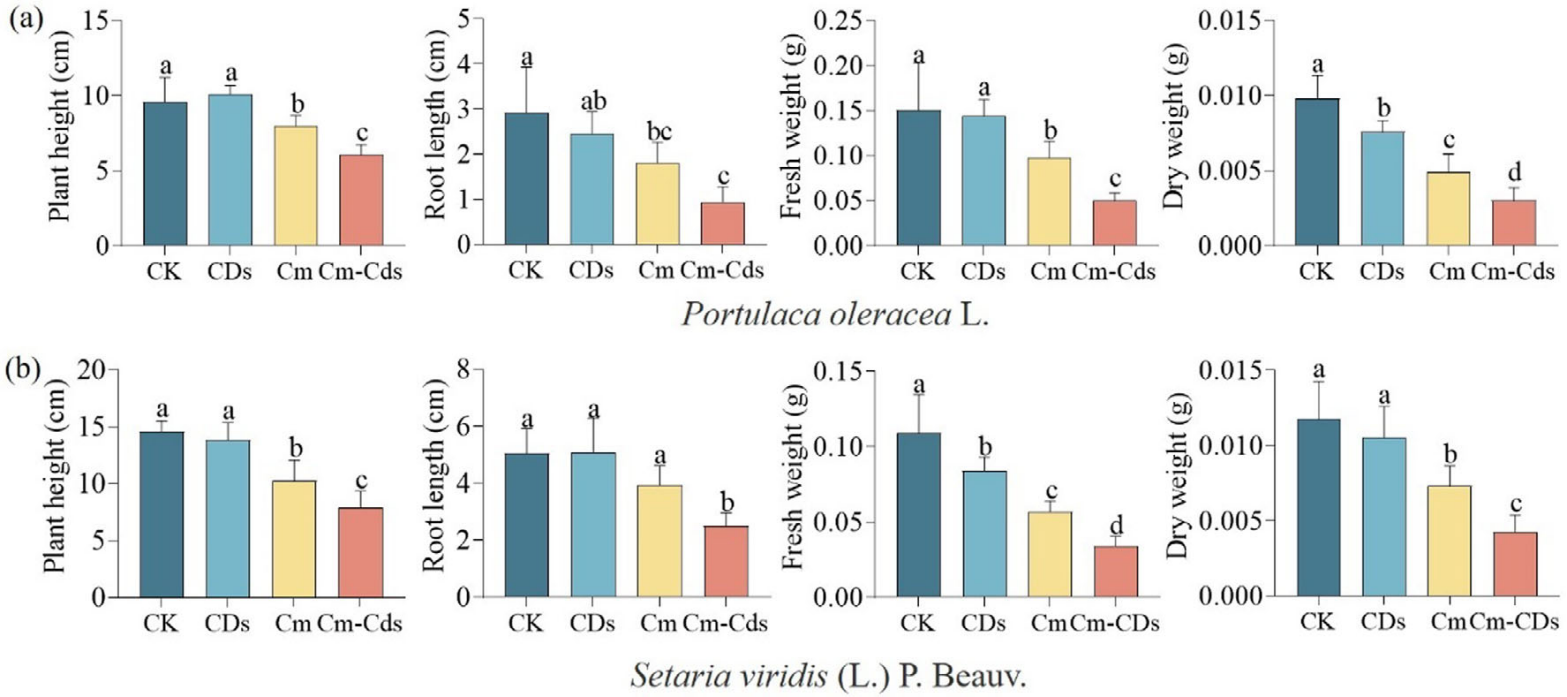
Determination of biomass of *Portulaca oleracea* (a) and *Setaria viridis* (b) after treatment with different agents at a concentration of 1.5 g/L.

Furthermore, analysis presented in Fig. 7 reveals that Cm‐CDs significantly influenced biomass parameters in both *P. oleracea* and *S. viridis* (*P* < 0.05). Compared to coumarin alone, Cm‐CDs increased the inhibition rates of *P. oleracea* by 19.59% in plant height, 29.45% in root length, 31.48% in fresh weight, and 18.9% in dry weight. Similarly, for *S. viridis*, the inhibition rates increased by 20.03% in plant height, 17.56% in root length, 21.09% in fresh weight, and 25.85% in dry weight.

### 3.4 Determination of toxicity of coumarin to weeds

To accurately evaluate the inhibitory doses of coumarin and Cm‐CDs, we established a toxicity regression equation by fitting the logarithmic concentrations of both compounds against the root length inhibition rates. The results are presented in Table 1. A strong linear relationship was observed, with correlation coefficients of 0.993 and 0.991, respectively. The IC_50_ of coumarin on *S. viridis* is determined to be 45.7 mg/L, while for *P. oleracea* it is 72.5 mg/L.

**Table 1:** Toxicity of coumarin to root length of two seeds

Test weed	Regression equation	Median inhibitory concentration, IC_50_ (mg/L)
*Setaria viridis viridis* (L.) P. Beauv.	*y* = −2.620*x* + 4.265	45.7 ± 10.347
*Portulaca oleracea* L.	*y* = −3.841*x* + 8.932	72.5 ± 15.05

### 3.5 Effects of coumarin and coumarin‐loaded carbon dots (Cm‐CDs) treatments on physiological parameters of two weeds

The concentration of coumarin presented in Table 1 was used as the research parameter to evaluate its growth inhibition effects on the two weed species. The Cm‐CDs solution was prepared based on the calculated optimal mass ratio, ensuring that both the coumarin solution and the Cm‐CDs solution contained equivalent concentrations of effective coumarin. As previously established, the CDs solution does not significantly impact weed germination or growth, therefore, only the effects of coumarin and Cm‐CDs solutions on *P. oleracea* and *S. viridis* were measured.

**Figure 8:**
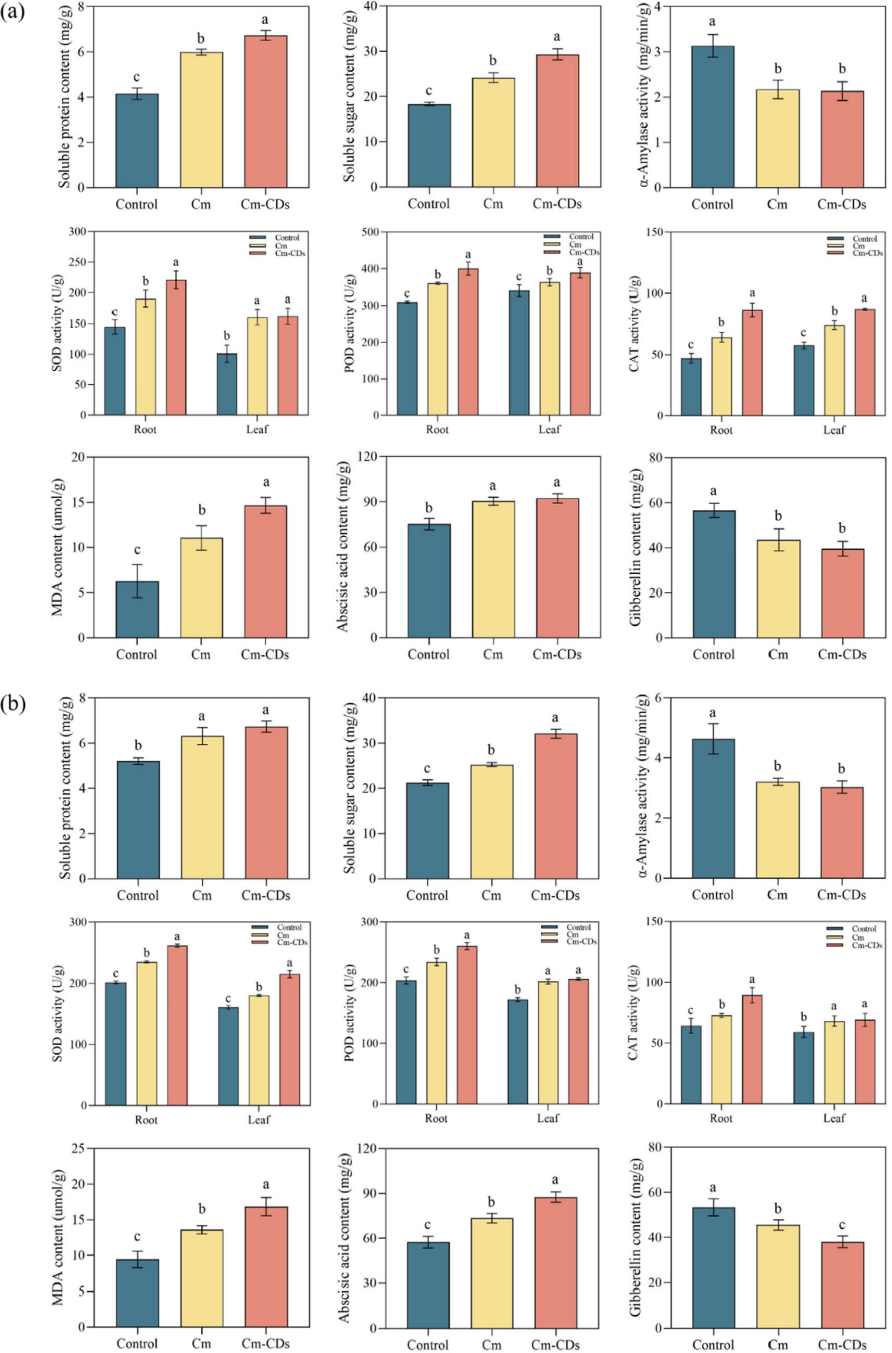
Effects of coumarin and coumarin‐loaded carbon dots (Cm‐CDs) treatment on soluble protein content, soluble sugar content, α‐amylase activity, superoxide dismutase (SOD) activity, peroxidase (POD) activity, catalase (CAT) activity, malondialdehyde (MDA) content, abscisic acid (ABA) content, and gibberellin (GA3) content in *Portulaca oleracea* (a) and *Setaria viridis* (b).

As shown in Fig. 8, treatment with coumarin and Cm‐CDs resulted in a significant increase in soluble protein content in *P. oleracea* (*P* < 0.05), with Cm‐CDs exhibiting a more pronounced effect (*P* < 0.05). The increases were 49.2% and 87.3%, respectively. Similarly, after treatment with coumarin and Cm‐CDs, *S. viridis* also exhibited a significant rise in soluble protein content (*P* < 0.05), with increases of 17.3% and 30.1%, respectively, compared to the control.

Additionally, both *P. oleracea* and *S. viridis* exhibited a notable increase in soluble sugar content following treatment with coumarin and Cm‐CDs (*P* < 0.05). In *P. oleracea*, the soluble sugar levels after treatment exceeded those of the control group by 38.3% and 72.2%. For *S. viridis* treated with coumarin and Cm‐CDs, the increases were 25.2% and 65.4%, respectively, compared to the control.

After treatment with coumarin and Cm‐CDs, the α‐amylase activity in *P. oleracea* and *S. viridis* was significantly lower than that of the control group (*P* < 0.05), indicating that both coumarin and Cm‐CDs effectively inhibit α‐amylase activity. This inhibition also negatively impacts seed germination.^29^ Although Cm‐CDs exhibited a stronger inhibitory effect, the difference compared to coumarin was not statistically significant (*P* > 0.05).

Following treatment with coumarin and Cm‐CDs, the activities of SOD, POD, and CAT in the root and leaf tissues of *P. oleracea* and *S. viridis* were significantly elevated compared to the control group (*P* < 0.05). Notably, the stress effect of Cm‐CDs on root tissues was markedly stronger than that of coumarin (*P* < 0.05). The inhibitory rates of Cm‐CDs on root activity in *P. oleracea* and *S. viridis* were 25.46% and 15.52% stronger than coumarin, respectively, suggesting a more pronounced inhibitory effect of Cm‐CDs on plant roots. Furthermore, the MDA content in both *P. oleracea* and *S. viridis* treated with coumarin or Cm‐CDs was significantly higher than that of the control group (*P* < 0.05). The increase in MDA content caused by Cm‐CDs was 57.4% and 34.74% greater than that of coumarin, respectively (*P* < 0.05).

After treatment with coumarin and Cm‐CDs, the ABA content in *P. oleracea* increased significantly compared to the control group (*P* < 0.05), but there was no significant difference between the coumarin and Cm‐CDs treatment groups. In *S. viridis*, the ABA content increased significantly after treatment with coumarin and Cm‐CDs (*P* < 0.05), and the effect of Cm‐CDs treatment was 19.19% higher than that of coumarin (*P* < 0.05). Additionally, the GA3 content in *P. oleracea* decreased significantly after treatment with coumarin and Cm‐CDs (*P* < 0.05). In *S. viridis*, the GA3 content also showed a significant decrease after treatment with coumarin and Cm‐CDs (*P* < 0.05), with the effect of Cm‐CDs being 16.41% higher than that of coumarin (*P* < 0.05).

### 3.6 Effects of coumarin and coumarin‐loaded carbon dots (Cm‐CDs) treatments on root development of two weeds

**Figure 9:**
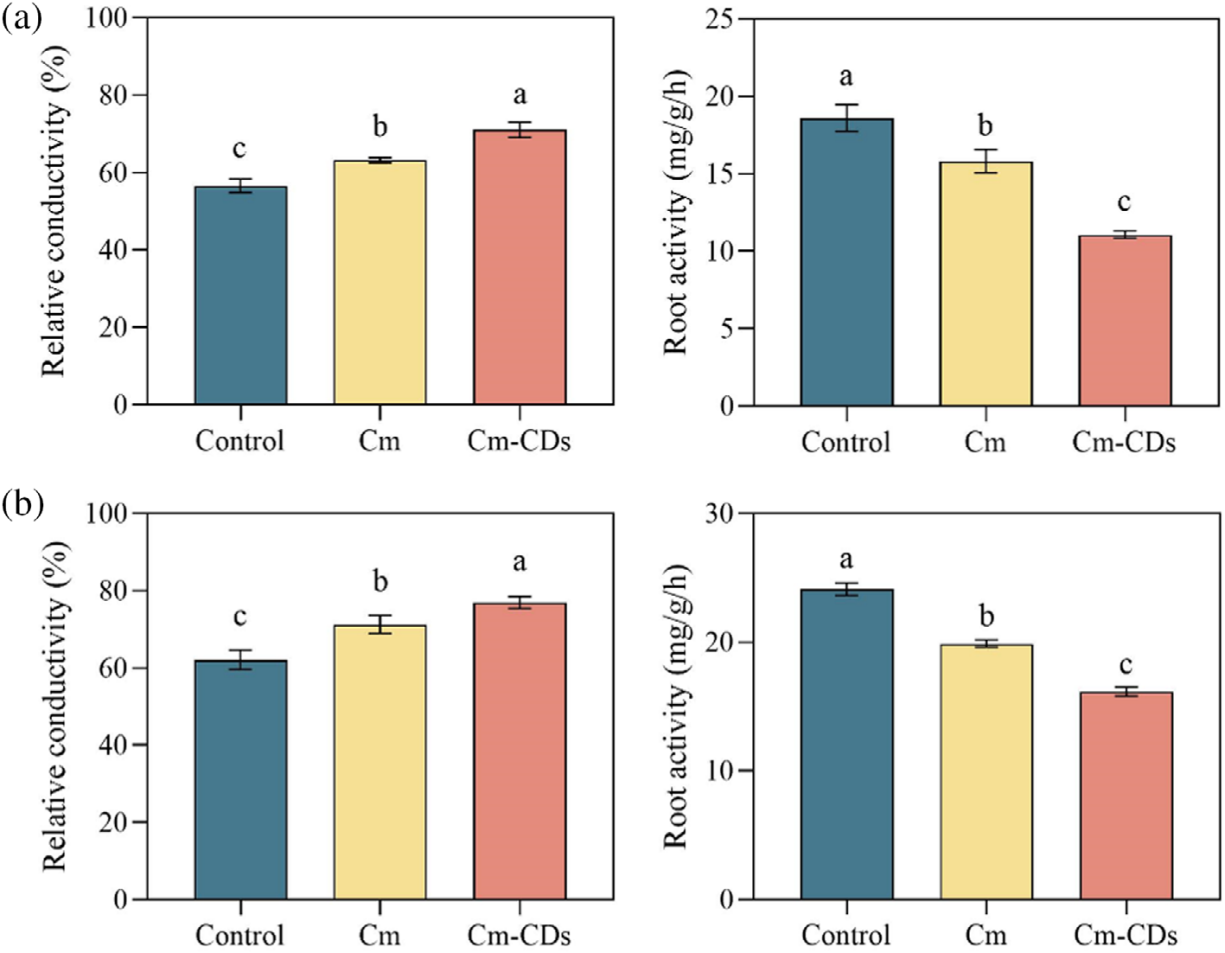
Effects of coumarin and coumarin‐loaded carbon dots (Cm‐CDs) treatments on root relative conductivity and root activity of *Portulaca oleracea* (a) and *Setaria viridis* (b).

As shown in Fig. 9, both coumarin and Cm‐CDs significantly increased the relative conductivity of *P. oleracea* and *S. viridis* (*P* < 0.05). The impact on root system relative conductivity was particularly pronounced (*P* < 0.05), with increases of 11.3% and 10.41%, respectively.

Additionally, both coumarin and Cm‐CDs markedly inhibited the root activity of *P. oleracea* and *S. viridis* (*P* < 0.05). Notably, Cm‐CDs demonstrated a more pronounced effect on root activity compared to coumarin alone. The inhibitory effects were significant (*P* < 0.05), with increases of 22.64% and 34.21%, respectively.

**Figure 10:**
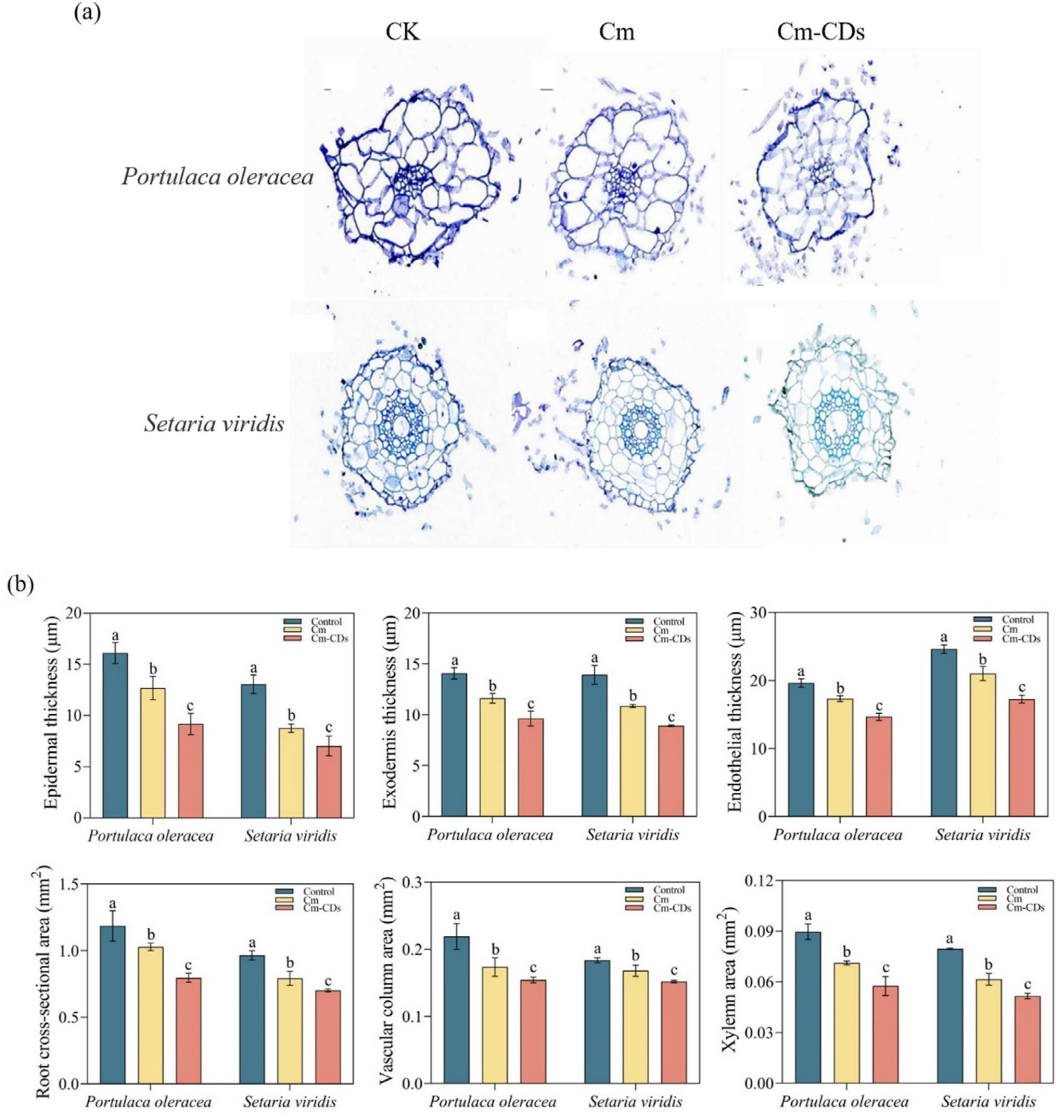
(a) Root cross‐section anatomical structure and (b) root anatomical structure parameters of *Portulaca oleracea* and *Setaria viridis* after treatment with coumarin and coumarin‐loaded carbon dots (Cm‐CDs).

Figure 10 illustrates the anatomical structure of the root cross‐section of *P. oleracea* and *S. viridis* following treatment with coumarin and Cm‐CDs. Coumarin and Cm‐CDs have been shown to adversely affect the root cells of *S. viridis* and *P. oleracea*. The plant cells treated with water exhibited a well‐organized structure, characterized by their large size and clearly defined cell walls. In contrast, the morphology of the cells exposed to coumarin was significantly compromised, resulting in deformed and loose cell walls.

The root cross‐sectional analysis of *P. oleracea* treated with Cm‐CDs revealed more pronounced cellular damage, featuring reduced cell sizes and incomplete, loosely organized individual cell walls. Similarly, the transversal epidermal cell layer of *S. viridis* roots subjected to Cm‐CDs treatment displayed extensive damage. The integrity of the cell wall was compromised, with observable black particulate matter present within both the cell wall and intercellular spaces of the root samples.

Quantitative analysis of measured parameters demonstrated significant reductions (*P* < 0.05) in epidermal thickness, exocortical thickness, endothelial thickness, root cross‐sectional area, vascular column area, and xylem area for both species following coumarin and Cm‐CDs treatments compared to controls. Notably, Cm‐CDs treatment resulted in greater reductions than coumarin alone. For *P. olerace*a, Cm‐CDs treatment caused significant decreases (*P* < 0.05) of 8.62% in epidermal thickness, 18.32% in exocortical thickness, 15.83% in endothelial thickness, 27.78% in root cross‐sectional area, 13.6% in vascular column area, and 18.5% in xylem area compared to coumarin treatment. Similarly, *S. viridis* exhibited reductions of 21.45% in epidermal thickness, 26.31% in exocortical thickness, 17.64% in endothelial thickness, 29.53% in root cross‐sectional area, 30.12% in vascular column area, and 38.02% in xylem area when comparing Cm‐CDs to coumarin treatment (*P* < 0.05).

Additionally, black granular material was observed within the cell walls and intercellular spaces at the roots of the samples examined. These findings collectively indicate that Cm‐CDs can compromise both cell wall integrity and permeability in weed roots, this alteration facilitates easier penetration into plant tissues from their roots while exerting a growth‐inhibiting effect.

## 4 DISCUSSION

Although numerous laboratory studies have demonstrated the potential application of coumarin in the agricultural field, its practical implementation in field settings remains constrained. This limitation primarily stems from coumarin's mode of action, which involves growth inhibition rather than direct lethality against target organisms. Consequently, this mechanism may necessitate increased application frequency and higher dosage requirements compared to conventional chemical herbicides. Nevertheless, these challenges do not negate coumarin's potential for field application. The primary research focus currently centers on enhancing its biological efficacy and improving application efficiency to facilitate practical implementation. The integration of advanced nanocarriers with plant‐derived pesticides presents an effective strategy for improving the performance of these natural products.^30^ Nanotechnology is increasingly recognized as a viable pathway for sustainable agricultural development.^31^ Experimental researches conducted on various plant species have demonstrated that nanocarriers can significantly improve the absorption and translocation of herbicide active ingredients within plants.^32^

Nanoparticle CDs exhibit excellent biodegradability and biocompatibility, characterized by rapid cellular absorption and high stability, making them suitable candidates for drug loading and release applications.^33^ The biological origin of CDs provides several advantages, including wide availability, low production costs, health benefits, and environmental safety, rendering them virtually harmless to humans and other organisms. In comparison to other nanomaterials, carbon‐based nanomaterials possess more stable properties that are less influenced by particle size variations,^34^ exhibiting superior characteristics. Therefore, biomass CDs emerge as ideal nanomaterials for enhancing both the activity and utilization efficiency of plant‐derived active substances. Based on these considerations, we selected black wolfberry as our raw material and prepared biomass CDs using a hydrothermal method. Black wolfberry is abundant in protein, fat, dietary fiber along with various vitamins and phenolic compounds, thus it serves as an excellent biomass raw material source rich in carbon content.

At present, the methods for synthesizing CDs typically include microwave radiation, hydrothermal processes, and laser ablation techniques. Among these, the hydrothermal method stands out for its environmental friendliness, cost‐effectiveness, and operational simplicity.^35^ This method has been successfully employed to prepare novel carbon‐based materials from various biomass feedstocks such as papaya, garlic and rice husk.^36^, ^37^, ^38^ CDs synthesized via the hydrothermal method exhibit excellent performance and are amenable to modification, facilitating their application in cellular and biological contexts. In addition to enabling the efficient introduction of drug molecules into biological cells, they can also serve as effective agents for biological imaging.^33^ After evaluating the advantages and limitations of several synthesis methods, this study concluded that the hydrothermal technique is particularly suitable for preparing CDs from black wolfberry. The CDs produced through this method generally demonstrate good hydrophilicity and high stability. Our characterization results support this conclusion: TEM revealed that the prepared CDs were uniformly distributed with a nearly spherical morphology. These CDs exhibited uniform dispersion (Fig. 3(b)). Infrared spectroscopy analysis indicated the presence of numerous hydrophilic functional groups on their surfaces, including carboxyl, hydroxyl, and amino groups, making them ideal nanocarriers for applications in plant systems (Fig. 3(d)).

Subsequently, we successfully assembled coumarin onto the surface of this complex, creating an optimized Cm‐CDs nanocomposite system. It has been reported that as the particle size of carbon nanomaterials decreases, the proportion of atoms at the outermost surface layer increases relative to the total number of atoms.^34^ This phenomenon, coupled with the lack of adjacent binding atoms around these surface atoms, leads to the formation of numerous unsaturated bonds that readily combine with other atoms while maintaining stability post‐combination. Consequently, this results in remarkably high chemical reactivity.^39^ Our study further confirmed that the biomass CDs and coumarin combine effectively, achieving an impressive drug loading rate of 65.45%.

We compared the effects of Cm‐CDs, coumarin, CDs, and deionized water (CK) on weed germination and growth under laboratory conditions. The results revealed that CDs exhibited excellent biocompatibility and minimal toxicity,^40^ showing no adverse effects on the growth and development of *P. oleracea* and *S. viridis*, nor any significant growth‐promoting effects. In contrast, both coumarin and Cm‐CDs demonstrated substantial inhibitory effects on weed germination and growth, with Cm‐CDs showing significantly enhanced efficacy (Fig. 5).

At a concentration of 60 mg/L, Cm‐CDs completely inhibited the germination of *S. viridis*. Although Cm‐CDs did not entirely prevent the germination of *P. oleracea* seeds, they effectively arrested post‐germination growth, resulting in wilting and mortality of all seedlings within 7–10 days. This phenomenon may be attributed to the rapid germination rate of *P. oleracea* seeds, which typically sprout within 2 days of cultivation, preceding the onset of the agent's activity. Following germination, Cm‐CDs effectively halted further growth, leading to gradual plant mortality. Moreover, for *P. oleracea*, the efficacy of Cm‐CDs at 100 mg/L was comparable to that of coumarin at 160 mg/L. This enhanced performance can be attributed to the CDs nanocarrier system, which facilitates improved penetration into plant tissues and cellular absorption, thereby increasing overall efficacy. In subsequent pot spraying tests conducted 1 week after applying coumarin and Cm‐CDs, visible inhibition in growth was observed for both *P. oleracea* and *S. viridis*, characterized by reduced plant height, leaf yellowing, and diminished biomass. Compared to coumarin alone, treatment with Cm‐CDs resulted in an increased inhibition rate for plant height (19.59%), root length (29.45%), fresh weight (31.48%), and dry weight (18.9%) in *P. oleracea*. Similarly notable increases were recorded for plant height (20.03%), root length (17.56%), fresh weight (21.09%), and dry weight (25.85%) in *S. viridis*. The experimental results indicate that we have successfully enhanced the biological activity and utilization efficiency of coumarin by integrating it with CDs.

Numerous successful reports highlight the application of nanocarriers in conjunction with natural compounds to form complexes for agricultural purposes. For instance, Yan *et al*. synthesized a simple, cost‐effective star‐shaped polymeric cation capable of stably binding to matrine through hydrogen bonding and hydrophobic interactions, resulting in the formation of a nanocomplex (10 nm).^41^ This approach not only improves the physical and chemical properties of matrine but also enables efficient transport to insect cells, enabling rapid action and thereby increasing its toxicity and duration of effect. Consequently, this method can effectively manage pests that are challenging to control in organic vegetable production, such as western flower thrips and aphids. Liang *et al*.^42^ employed an *in situ* crystal growth strategy to synthesize ZnO‐Z nanospheres with a core‐shell structure for berberine (Ber) loading, achieving effective sterilization while significantly mitigating damage caused by tomato bacterial wilt. These studies collectively provide valuable insights into the development of precise and efficient pest control strategies using plant‐derived bioactive substances.

To further validate that the combination of coumarin with CDs nanocomplexes maintains its original mode of action and safety profile, we conducted a comparative analysis of the physiological and biochemical effects of Cm‐CDs and coumarin on plants. We established the concentration of coumarin at its IC_50_ level and treated weeds with a solution of Cm‐CDs combined with an equivalent amount of coumarin. This experimental design enabled us to simultaneously assess growth inhibition, survival, and growth through a comprehensive series of physiological indices.

The results indicated that the levels of soluble protein and soluble sugar in *P. oleracea* and *S. viridis* following treatment with coumarin and Cm‐CDs were significantly higher than those in the control group, however, the effect observed with Cm‐CDs was markedly stronger. Additionally, α‐amylase activity across all weed species exhibited a significant decrease, which was more pronounced in plants treated with Cm‐CDs. These findings suggest that both Cm‐CDs and coumarin exert similar effects on weeds by inhibiting root length and seedling length, nevertheless, the rate of inhibition is enhanced when coupled with CDs nanoparticles. This enhancement facilitates improved penetration of coumarin into plant tissues, thereby increasing its effectiveness in disrupting protein and sugar utilization.

Further analysis revealed variations in phytohormone content within *P. oleracea* and *S. viridis*. Notably, GA3 levels decreased more significantly after treatment with Cm‐CDs compared to those treated solely with coumarin, while ABA levels increased more substantially under similar conditions (Fig. 8). The elevated ABA levels stimulate the production of storage proteins and fats in seeds, induce embryonic cells to cease division, and initiate and maintain seed dormancy, while simultaneously inhibiting seedling growth and development. Conversely, GA3 promotes the release from seed dormancy as well as germination and growth. Our findings further confirm that Cm‐CDs can more effectively prevent plants from utilizing proteins and sugars, thereby impacting material and energy metabolism during seed germination. This disruption leads to insufficient energy supply during this critical phase, ultimately achieving effective inhibition of both seed germination and subsequent plant growth.

Furthermore, our investigation demonstrated that Cm‐CDs treatment elicited significantly higher activities of key antioxidant enzymes such as SOD, POD, and CAT, compared to coumarin treatment alone, with the exception of SOD activity in *P. oleracea* leaves and POD/CAT activities in *S. viridis* leaves. This suggests that both *P. oleracea* and *S. viridis* have experienced stress due to exogenous treatments, triggering a robust increase in protective enzyme production as a defense mechanism against oxidative stress. The notable rise in MDA content further indicates severe oxidative damage sustained by these plants.^43^ These observations imply that the stress inflicted by Cm‐CDs on the membrane structure and cellular integrity of weeds is greater than that caused by coumarin treatment.

As a crucial interface between plants and soil, plant roots serve as the primary organs for the absorption and transportation of water and nutrients, exhibiting heightened sensitivity to allelochemicals present in the soil.^44^ Root activity and electrical conductivity are vital indicators of root health.^45^ Our findings reveal that both coumarin and Cm‐CDs significantly impair root function in the tested plants, leading to a significant reduction in root activity while concurrently increasing their relative electrical conductivity. Notably, Cm‐CDs exhibited more pronounced effects compared to coumarin alone. Allelopathic substances primarily exert their stress effects on root systems. When subjected to such stress, roots typically initiate morphological adaptations as a protective response.^46^ According to our anatomical analysis of the root system presented in this study, coumarin and Cm‐CDs exhibited a negative effect on the cell morphology and cell wall structure of the two weed species. Notably, the cells treated with Cm‐CDs experienced significantly greater damage. Specifically, *P. oleracea* roots treated with Cm‐CDs showed markedly compromised cell wall integrity compared to controls (Fig. ure 10), exhibiting loosened structures with complete loss of integrity in some individual cell walls. Quantitative analysis revealed significant reductions (*P* < 0.05) in multiple root parameters under Cm‐CDs treatment compared to coumarin alone, including epidermal thickness, exocortical thickness, endothelial thickness, root cross‐sectional area, vascular column area, and xylem area. These findings indicate that Cm‐CDs treatment induces more substantial root morphological modifications as a stress response mechanism.

Our comprehensive analysis revealed comparable physiological and biochemical activities between coumarin and Cm‐CDs, yet Cm‐CDs demonstrated superior weed suppression efficacy. Previous studies suggest that CDs primarily penetrate plant tissues through cell wall pores. The waxy hydrophobic cuticle of plants contains nanopores averaging approximately 5 nm in diameter,^47^ while our synthesized CDs measured about 5.71 nm. This dimensional similarity, combined with the large specific surface area of CDs, suggests that when loaded with coumarin, these nanoparticles can more effectively penetrate root cell walls and utilize passive diffusion for efficient upward transport. This mechanism facilitates more stable coumarin delivery into plant systems, where it exerts enhanced allelopathic effects, resulting in more rapid and potent growth inhibition.

## 5 CONCLUSION

Our investigations revealed that the black wolfberry CDs solution did not adversely affect the growth of *P. oleracea* and *S.viridis*. However, when combined with CDs nanocarriers, there was a significant enhancement in the herbicidal biological activity of coumarin. At lower concentrations, the Cm‐CD complex effectively inhibited seed germination in *S. viridis* and arrested post‐germination growth in *P. oleracea*. Notably, at a spray concentration of 1.5 g/L, the formulation completely prevented normal plant growth and development, ultimately leading to plant wilting.

Both Cm‐CDs and coumarin demonstrated similar modes of action, including inhibition of weed biomass accumulation, alteration of hormone levels, modification of root activity and morphology, as well as enhancement of antioxidant enzyme activity and MDA content. Importantly, Cm‐CDs consistently exhibited superior efficacy compared to coumarin alone across all evaluated parameters. These findings highlight the dual role of CDs as effective nanocarriers that not only enhance the absorption rate but also potentiate the biological activity of coumarin in weed control, thereby validating their potential as nanopesticide carriers.

While the current data were obtained under controlled laboratory conditions, our future research will focus on exploring the application methods and field efficacy of Cm‐CDs, aiming to develop more effective solutions for agricultural practices. Further field studies are warranted to fully assess the practical implications of this nanotechnology‐based approach in pest management.

## AUTHOR CONTRIBUTIONS

Conceptualization: YYang; methodology, software: JZ; validation: CW; formal analysis: Y Yin; investigation, resources: ZC; data curation, writing – original draft preparation, Y. Yang and YYin; writing – review and editing: CW; visualization: YYang and YYin; supervision, project administration: CW. All authors have read and agreed to the published version of the article.

## CONFLICT OF INTEREST STATEMENT

The authors declare that they have no known competing financial interests or personal relationships that could appear to influence the work reported in this article.

## Data Availability

The data that support the findings of this study are available from the corresponding author upon reasonable request.

## References

[1] Duke SO , Glyphosate: environmental fate and impact. Weed Science 68:201–207 (2020).

[2] Wang J , Hou J , Wang L , Zhu Z , Han B , Chen L *et al*., Pollution characteristics, environmental issues, and green development of neonicotinoid insecticides in China: insights from imidacloprid. Environ Pollut 365:125394 (2024). 39586452 10.1016/j.envpol.2024.125394

[3] Henriquez J , Domoradzki J , Yan Z , Papineni S , Gehen S , Murphy L *et al*., Agricultural Triazole Fungicides. Patty's Toxicology pp. 1–31 (2023).

[4] Zhangqian W , Jun‐Ran K , Mo W , Shaohua S and Young‐Joon A , Larvicidal activity of Cnidium monnieri fruit coumarins and structurally related compounds against insecticide‐susceptible and insecticide‐resistant Culex pipiens pallens and Aedes aegypti. Pest Manag Sci 68:1041–1047 (2012). 22389164 10.1002/ps.3265

[5] SN V , AV A , DS L and JB S , Synthesis and biological evaluation of coumarin appended thiazole hybrid heterocycles: antibacterial and antifungal study. J Mol Struct 1293:136229 (2023). 10.1016/j.molstruc.2023.136229.

[6] Nazemi A‐H , Asadi GA and Ghorbani R , Herbicidal activity of coumarin when applied as a pre‐plant incorporated into soil. Not Sci Biol 7:239–243 (2015).

[7] Liu J , Zhou H , Guo F and Ding W , Research progress on pesticide activity of coumarins and acaricidal mechanism of scopoletin. J Pest Sci 21:692–708 (2019).

[8] Yuan X , Yang F , Wang Y , Li S , Zhang D , Liang W *et al*., Scopoletin negatively regulates the HOG pathway and exerts antifungal activity against Botrytis cinerea by interfering with infection structures, cell wall, and cell membrane formation. Phytopathology Research 6:1–1 (2024).

[9] Zaynab M , Fatima M , Abbas S , Sharif Y , Umair M , Zafar MH *et al*., Role of secondary metabolites in plant defense against pathogens. Microb Pathog 124:198–202 (2018). 30145251 10.1016/j.micpath.2018.08.034

[10] Nazemi AH , Asadi GA and Ghorbani R , Allelopathic potential of Lavender's extract and coumarin applied as pre‐plant incorporated into soil under agronomic conditions. Planta Daninha 36 (2018).

[11] Ghofrane J , ZJ G and Rabiaa H , Allelochemicals from Thapsia garganica leaves for Lolium perenne L. control: the magic of mixtures. Chem 32:81–87 (2022).

[12] Johnston JJ , Introduction of new pesticides. Pest Sci 4:46 (2004).

[13] Sun G , Liu Y , Deng J and Yuan H , Field experiment on controlling wheat powdery mildew with plant pesticide osthol. Hubei plant protection 3:6–7 (2016).

[14] Bijoy G and Sangeetha D , Biomass derived carbon quantum dots as potential tools for sustainable environmental remediation and eco‐friendly food packaging. J Environ Chem Eng 12:113727 (2024).

[15] Gao N , Yang W , Nie H , Gong Y , Jing J , Gao L *et al*., Turn‐on theranostic fluorescent nanoprobe by electrostatic self‐assembly of carbon dots with doxorubicin for targeted cancer cell imaging, in vivo hyaluronidase analysis, and targeted drug delivery. Biosens Bioelectron 96:300–307 (2017). 28511113 10.1016/j.bios.2017.05.019

[16] Dong J , Wang Q , Gu T , Liu G , Petrov YV , Baulin VE *et al*., Rapamycin functionalized carbon dots: target‐oriented synthesis and suppression of vascular cell senescence. J Colloid Interface Sci 660:534–544 (2024). 38266335 10.1016/j.jcis.2024.01.032

[17] Wang Z , Li Y , Zhang B , Gao X , Shi M , Zhang S *et al*., Functionalized carbon dot‐delivered RNA Nano fungicides as superior tools to control phytophthora pathogens through plant RdRP1 mediated spray‐induced gene silencing. Adv Funct Mater 33:2213143 (2023). 10.1002/adfm.202213143.

[18] Biswajit G , Soubantika P and Joydeep C , Carbon dots: a Mystic star in the world of nanoscience. Journal of Nanomaterials 2019:1–19 (2019).

[19] Wang X and Xing S , Study on determination of protein content by coomassie brilliant blue method. Tianjin chemical industry 23:40–42 (2009).

[20] Ding X , Zhang X , Zhao Y , Tan Z , Li Y , Wang P *et al*., Improved test method for determination of soluble sugar content by anthrone colorimetric method. Heilongjiang Animal Husbandry and Veterinary Medicine 12:230–233 (2014).

[21] Guo D and Peng X , Comparative study on two methods of starch determination by anthrone colorimetric method and enzymatic hydrolysis method. Journal of Hunan University of Arts and Sciences 19:34–36 (2007).

[22] Qu M , Qin L , Liu Y , Fan H , Zhu Z and Wang J , Comparison of two methods for detecting SOD activity. Journal of Food Safety and Quality Inspection 5:3318–3323 (2014).

[23] Li Z and Gong M , Improvement of determination of plant peroxidase activity by guaiacol method. Plant physiology communication 44:323–324 (2008).

[24] Gordo SG , Ruiz MR , Palma JM and Corpas FJ , Comparative analysis of catalase activity in plants: spectrophotometry and native PAGE approaches. Methods Mol Biol 2798:213–221 (2024). 38587746 10.1007/978-1-0716-3826-2_15

[25] Zhang Q and Zhang Y , Response of malondialdehyde (MDA) content in plants to drought. Forestry exploration and design 48:110–112 (2019).

[26] Mavi K , Mavi FL , Demir I and Matthews S , Electrical conductivity of seed soak water predicts seedling emergence and seed storage potential in commercial seed lots of radish. Seed Science and Technology 42:76–86 (2014).

[27] Yan Z‐Q , Tan J , Guo K and Yao L‐G , Phytotoxic mechanism of allelochemical liquiritin on root growth of lettuce seedlings. Plant Signal Behav 15:1795581 (2020). 32693669 10.1080/15592324.2020.1795581 PMC8550531

[28] Baishya N , Bora N , Athparia M , Padhi P and Kataki R , Hydrothermal conversion of biomass for co‐production of carbon quantum dots and biofuels. Environ Sci Pollut Res 1–15 (2025). 10.1007/s11356-024-35842-x 39751683

[29] Yang N , He X , Ran L , Yang F , Ma C , Chen H *et al*., The mechanism of coumarin inhibits germination of ryegrass (Lolium perenne) and its application as coumarin‐carbon dots nanocomposites. Pest Manag Sci 79:2182–2190 (2023). 36740923 10.1002/ps.7397

[30] Huang X , Ni X , Li H , Wei Y , Wang Z , Zhen C *et al*., Synergistic mechanism of botanical pesticide camptothecin encapsulated in a nanocarrier against fall armyworm: enhanced stability and amplified growth suppression. Ecotoxicol Environ Saf 284:116900 (2024). 10.1016/j.ecoenv.2024.116900. 39168084

[31] Gomollón‐Bel F , Ten chemical innovations that will change our world: IUPAC identifies emerging technologies in chemistry with potential to make our planet more sustainable. Chem Int 41:12–17 (2019).

[32] Ni Y , H X , R L , Y F , M C , C H *et al*., The mechanism of coumarin inhibits germination of ryegrass (Lolium perenne) and its application as coumarin‐carbon dots nanocomposites. Pest Manag Sci 79:2182–2190 (2023). 10.1002/ps.7397. 36740923

[33] Meng W , Bai X , Wang B , Liu Z , Lu S and Yang B , Biomass‐derived carbon dots and their applications. Energy & Environmental Materials 2:172–192 (2019).

[34] Alivisatos AP , Gu W and Larabell C , Quantum dots as cellular probes. Annu Rev Biomed Eng 7:55–76 (2005). 16004566 10.1146/annurev.bioeng.7.060804.100432

[35] Kumar P , Dua S , Kaur R , Kumar M and Bhatt G , A review on advancements in carbon quantum dots and their application in photovoltaics. RSC Adv 12:4714–4759 (2022). 35425490 10.1039/d1ra08452f PMC8981368

[36] Kasibabu BSB , D'souza SL , Jha S and Kailasa SK , Imaging of bacterial and fungal cells using fluorescent carbon dots prepared from Carica papaya juice. J Fluoresc 25:803–810 (2015). 26123674 10.1007/s10895-015-1595-0

[37] Shaojing Z , Minhuan L , Xiaoyue Z , Hongtao X , Tsz‐Wai N , Xiangmin M *et al*., Green synthesis of bifunctional fluorescent carbon dots from garlic for cellular imaging and free radical scavenging. ACS Appl Mater Interfaces 7:17054–17060 (2015). 26193082 10.1021/acsami.5b03228

[38] Zhaofeng W , Jingfang Y , Xin Z , Na L , Bin L , Yanyan L *et al*., Large‐scale and controllable synthesis of graphene quantum Dots from Rice husk biomass: a comprehensive utilization strategy. ACS Appl Mater Interfaces 8:1434–1439 (2016). 26710249 10.1021/acsami.5b10660

[39] Yang Z , Preparation, Characterization and Application of biomass carbon points in analysis and detection [Master] (2018).

[40] Maholiya A , Ranjan P , Khan R , Murali S , Nainwal R , Chauhan PS *et al*., An insight into the role of carbon dots in agriculture system: a review. Environ Sci Nano (2023).

[41] Yan S , Hu Q , Li J , Chao Z , Cai C , Yin M *et al*., A star polycation acts as a drug nanocarrier to improve the toxicity and persistence of botanical pesticides. ACS Sustainable Chemistry & Engineering 7:17406–17413 (2019).

[42] Liang W , Cheng J , Zhang J , Xiong Q , Jin M and Zhao J , pH‐responsive on‐demand alkaloids release from core–shell ZnO@ZIF‐8 nanosphere for synergistic control of bacterial wilt disease. ACS Nano 16:2762–2773 (2022). 35135193 10.1021/acsnano.1c09724

[43] Hodges DM , DeLong JM , Forney CF and Prange RK , Improving the thiobarbituric acid‐reactive‐substances assay for estimating lipid peroxidation in plant tissues containing anthocyanin and other interfering compounds. Planta 207:604–611 (1999). 10.1007/s00425-017-2699-3 28456836

[44] Yan SB and Wang P , Effects of alleolchemicals on morphological traits of roots: a meta‐analysis. Ying Yong Sheng Tai Xue Bao 31:2168–2174 (2020). 32715678 10.13287/j.1001-9332.202007.001

[45] Liu JJ , Wei Z and Li JH , Effects of copper on leaf membrane structure and root activity of maize seedling. Bot Stud 55:1–6 (2014). 28510936 10.1186/s40529-014-0047-5 PMC5432969

[46] Oh E , Seo PJ and Kim J , Signaling peptides and receptors coordinating plant root development. Trends Plant Sci 23:337–351 (2018). 29366684 10.1016/j.tplants.2017.12.007

[47] Begum P and Fugetsu B , Phytotoxicity of multi‐walled carbon nanotubes on red spinach (Amaranthus tricolor L) and the role of ascorbic acid as an antioxidant. J Hazard Mater 243:212–222 (2012). 23146354 10.1016/j.jhazmat.2012.10.025

